# Etymologia: *Opisthorchis*

**DOI:** 10.3201/eid2001.ET2001

**Published:** 2014-01

**Keywords:** etymologia, Opisthorchis, trematodes, flatworms, flukes

***Opisthorchis* [oʺpis-thorʹkis]**

From the Greek *opisthen* (behind) and *orchis* (testicle), *Opisthorchis* is a genus of trematode flatworms whose testes are located in the posterior end of the body. Rivolta is generally credited with discovering the first opisthorchid, which he named *Distoma felineus*, in a cat in Italy in 1884. However, the fluke may have been mentioned by Rudolphi in 1819, and in 1831, Gurlt published a textbook that included a drawing of a fluke that was almost certainly *Opisthorchis*. By the end of the 19th century, *Distoma* contained so many species that Blanchard introduced the genus *Opisthorchis* for elongated flat flukes with testes in the posterior end of the body. He chose Rivolta’s *Opisthorchis felineus* as the type species.
